# Efficacy and safety of immunotherapy in anaplastic thyroid carcinoma: a systematic review and meta-analysis

**DOI:** 10.1097/JS9.0000000000003301

**Published:** 2025-08-27

**Authors:** Kang Ning, Qiaorong Chen, Yu Guo, Hao Li, Bu Zou, Taonong Cai, Li Wang, Yongchao Yu, Zhenyu Luo, Jian Bu, Han Hong, Ziying Li, Xujia Wen, Mingjie Jiang, Tong Wu, Tianrun Liu, Weichao Chen, Zan Jiao, Ankui Yang

**Affiliations:** a Department of Head and Neck Surgery, Sun Yat-sen University Cancer Center, Guangzhou, China; b State Key Laboratory of Oncology in Southern China, Collaborative Innovation Center for Cancer Medicine, Guangzhou, China; c Zhongshan School of Medicine, Sun Yat-sen University, Guangzhou, China; d Department of Thyroid Center/Thyroid Surgery, The Sixth Affiliated Hospital, Sun Yat-sen University, Guangzhou, China; e Department of Otorhinolaryngology Head and Neck Surgery, The Sixth Affiliated Hospital, Sun Yat-sen University, Guangzhou, China; f Department of Thyroid Surgery, Sun Yat-sen Memorial Hospital, Sun Yat-sen University, Guangzhou, China; g The Second Clinical College of Hainan Medical University, Haikou, China; h Department of Medical Oncology, Sun Yat-sen University Cancer Center, Guangzhou, China; i School of Medicine, Shenzhen Campus of Sun Yat-sen University, Sun Yat-sen University, Shenzhen, China

**Keywords:** anaplastic thyroid carcinoma, immunotherapy, prognosis, systematic review

## Abstract

**Background:**

Anaplastic thyroid carcinoma (ATC) is a rare, aggressive solid tumor with poor prognosis. Traditional treatment such as surgery, radiotherapy, and chemotherapy has limited therapeutic effects. In recent years, immunotherapy is changing the treatment pattern of them. We systematically summarize the research status and application of immunotherapy in ATC to provide clinicians and relevant researchers with a comprehensive perspective.

**Method:**

A comprehensive search of the PubMed and Embase databases identified 18 studies investigating the application of immunotherapy in ATC. These included ten cohort studies and eight case reports. The quality of the included studies was assessed using the Newcastle-Ottawa Quality Assessment Scale (NOS) and Joanna Briggs Institute checklist (JBI). Subsequently, a single-proportion meta-analysis and meta-regression were performed to evaluate the efficacy of immunotherapy in ATC.

**Results:**

The favorable response rate to immunotherapy in ATC patients (33–100%) varies across cohorts, indicating substantial heterogeneity. The combination of immunotherapy with targeted therapy, as well as the use of neoadjuvant treatment, has led to improved outcomes for ATC. Single-proportion meta-analysis suggests that immunotherapy may provide clinical benefits for a subset of ATC patients (approximately one-third to one-half), but its effectiveness in controlling disease progression remains limited. Meta-regression further indicates that ATC patients with PD-L1 expression or BRAF^V600E^ mutations tend to have better treatment responses. Among the 8 case reports, two patients achieved complete remission after immunotherapy, and four patients died of disease progression after immunotherapy. Mild adverse effects are common, but interstitial pneumonia is associated with poor prognosis in immunotherapy.

**Conclusion:**

Immunotherapy for ATC has demonstrated safety and efficacy in several studies, especially in patients with PD-L1 expression or BRAF^V600E^ mutations. However, immune-related side effects should be carefully managed, particularly to prevent interstitial pneumonia.

**HIGHLIGHTS**

- This systematic review provided a valuable overview of the latest research status and application of immunotherapy and its combination therapies for ATC, comprehensively analyzing the efficacy and potential adverse events of various immunotherapy regimens.
- Early or locally advanced ATC patients can be considered for neoadjuvant immunotherapy followed by surgery. For ATC patients with BRAFV600E gene mutation and or PD-L1 positive expression, targeted therapy combined with immunotherapy can be used in a targeted manner.
- Emphasize the need for vigilant monitoring of adverse events related to immunotherapy, particularly interstitial pneumonia in patients with lung metastasis.

## Introduction

Our article is compliant with the TITAN Guidelines 2025 – governing declaration and use of AI^[1]^.

Anaplastic thyroid carcinoma (ATC) is a malignant tumor that originates from thyroid follicular cells, characterized pathologically by significant cellular atypia and a complete loss of differentiation capacity^[2]^. Although the incidence of ATC is relatively low, accounting for 1.3% to 9.8% of thyroid cancer cases, it is characterized by its highly invasive nature, rapid progression, and extreme malignancy^[3]^. Studies indicate that approximately 30–58% of patients already have distant metastases at the time of diagnosis, with a median survival of merely 5 months and a one-year survival rate as low as 20%^[3,4]^. The conventional treatment options for ATC, including surgery, radiotherapy, and chemotherapy, are relatively limited^[5]^. Although current targeted therapies, like Dabrafenib and Trametinib, have shown good clinical activity, their efficacy is limited to ATC cases with BRAF ^V600E^ mutations^[6]^. Moreover, even when targetable mutations are present, patients may develop new genetic mutations during treatment, leading to resistance to targeted drugs^[7]^.

Recent explorations into the application of immunotherapy are bringing new hope to this orphan disease. Immunotherapy works by activating or modulating the body’s immune system, enabling it to recognize and eliminate cancer cells, thereby combating tumor initiation and progression^[8]^. The strategies of modern immunotherapy have significantly expanded to include a variety of approaches, among which immune checkpoint inhibitors (ICIs) are particularly prominent, profoundly transforming cancer treatment paradigms^[9,10]^. The FDA first approved ipilimumab (anti-CTLA-4) for metastatic melanoma in 2011, followed by pembrolizumab and nivolumab^[11,12]^. Since then, ICIs-based combination immunotherapy has become a first-line treatment for several major types of cancer, including hepatocellular carcinoma, renal cell carcinoma, lung cancer, cervical cancer, and gastric cancer^[13–18]^. For example, pembrolizumab monotherapy show durable antitumor activity and manageable safety in a phase II study of triple-negative breast cancer^[19]^. In non-small cell lung cancer, immunotherapy combined therapy can significantly improve the progression-free survival (PFS) and overall survival (OS) comparing with chemotherapy^[20,21]^.

Compared with differentiated thyroid carcinoma (DTC), ATC exhibits a tumor immune microenvironment characterized by higher levels of tumor-infiltrating lymphocytes, significantly increased exhausted CD8^+^ T cells, and elevated PD-L1 expression^[22–24]^. These features suggest that ATC patients may have a favorable response to immunotherapy. Currently approved and marketed PD-1/PD-L1 inhibitors such as pembrolizumab and spartacizumab have achieved certain results in clinical trials^[25–27]^. A retrospective study showed that the combination of dabrafenib + trametinib with pembrolizumab resulted in an 8-month longer median OS and 3-month longer median PFS compared to the combination without pembrolizumab^[26]^. However, due to the highly aggressive nature of ATC, some clinical studies have not yielded satisfactory results. For instance, Chintakuntlawar *et al* conducted a clinical trial combining radiation therapy with pembrolizumab for ATC, but all three enrolled patients died within six months of treatment^[28]^. In addition to the heterogeneity of some study findings, there are other issues with clinical research on immunotherapy for ATC. Due to the rarity of ATC, individual studies often have insufficient sample sizes, which limits the stability and reliability of conclusions regarding immunotherapy for ATC. Therefore, it is necessary to consolidate existing research and conduct a more in-depth investigation into the precise efficacy and side effects of immunotherapy for ATC.

In this systematic review, we comprehensively examined the current state of immunotherapy research for ATC, including prospective and retrospective clinical studies as well as relevant case reports. By systematically organizing and analyzing these studies, we have provided a comprehensive summary of the application of immunotherapy in ATC, highlighting its current status, achievements, and challenges.

## Methods

### Protocol registration

This systematic review was performed in accordance with the Preferred Reporting Items for Systematic Reviews and Meta-Analyses (PRISMA) and Assessing the Methodological Quality of Systematic Reviews (AMSTAR) guidelines^[29,30]^. The protocol of this study has been registered with the International Prospective Register of Systematic Reviews prior to the initiation of data extraction.

### Search strategy

A search of PubMed and Embase databases for subject terms “anaplastic thyroid carcinoma”, “poorly differentiated thyroid carcinoma”, “undifferentiated thyroid carcinoma,” “immunotherapy”, “immune checkpoint inhibitor” were performed to retrieve articles published in reviews and clinical trials from the beginning of their inception until 31 December 2024. The detailed search strategy and process have been recorded in Supplemental Digital Content Table 1, available at: http://links.lww.com/JS9/E955.

### Eligible criteria

Studies were included if they met the following criteria: (1) Randomized controlled trials (RCTs), prospective and retrospective observational studies and case reports; (2) Reported associations of ATC patients with immunotherapy or immunotherapy combined with other treatment modalities; (3) The studies or case reports must include essential clinical information and provide either direct or indirect data on patient survival or treatment response; (4) ATC in human adults; (5) published in English journals.

Exclusion criteria were as follows (1): Non-original research, such as reviews, letters, comments and conference abstracts; (2) Only the efficacy of other treatments (such as targeted therapies) in patients with ATC was reported; (3) Studies lacking sufficient information to assess the effect of immunotherapy on the prognosis of ATC patients; (4) Unpublished data and those studies were also excluded if they were not published in peer-reviewed publications.

### Study selection and data extraction

After removing duplicates, all titles and abstracts underwent a preliminary screening. Full-text articles of potentially relevant studies were retrieved for further assessment of eligibility by two independent reviewers. Any discrepancies were resolved through discussions with a senior reviewer. Additionally, manual reference list searches of retrieved studies were conducted to identify additional eligible studies. Data extraction was performed according to a predefined data extraction form by two reviewers independently, and the results were further verified by a senior reviewer. The following information was extracted from each study: first author and publication year, study design, study population, sample size, outcome of interest, exposure assessment, and main findings. The outcome measures used to evaluate the efficacy of immunotherapy included complete response (CR), partial response (PR), stable disease (SD), progressive disease (PD), progression-free survival (PFS), overall survival (OS), and 1-year survival rate. When there were duplicate studies based on the same cohort, we prioritized the results with the larger sample size. The reviewers reached consensus on any discrepancies in data extraction.

### Study quality assessment

The NOS was employed to evaluate the quality of case-control and cohort studies based on three domains: selection of study groups (0–4 scores), comparability of groups (0–2 scores) and ascertainment of exposure or outcomes (0–3 scores)^[31–33]^. The total scores of 0–3, 4–6, and 7–9 were considered to represent low, moderate and high quality, respectively. To evaluate the quality of RCTs, the Cochrane risk of bias tool was employed, which assesses six domains, including sequence generation, allocation concealment, binding, incomplete outcome data, selective outcome reporting and other bias^[34]^. The studies were categorized as high, low or unclear risk of bias in each domain. Two investigators independently evaluated the studies, and disagreements were resolved by consensus. The case reports were evaluated using the JBI checklist^[35]^. Two investigators independently assessed the studies, and any disagreements were resolved through consensus.

### Statistical analysis

To thoroughly investigate the disease progression and survival outcomes of ATC patients following immunotherapy, we selected 10 cohort studies for single-proportion meta-analysis. The results were visually presented using funnel plots, forest plots and bubble charts. Heterogeneity among these studies was assessed by the Chi^2^ and I^2^ statistic. *P* value of Chi^2^ >0.05 or I^2^ <50% indicated the absence of heterogeneity, then a fixed effects model was selected to estimate the results^[36]^. Otherwise, the random effects model was selected, which provided more conservative results than a fixed effects model^[37]^. Publication bias was visually assessed using funnel plots^[38]^. Meta-regression was performed for selected variables to identify their potential impact on the efficacy of immunotherapy. All data were analyzed in R software (version 4.5.1) using the meta package (https://CRAN.R-project.org/package=meta), and *P* values <0.05 were considered statistically significant for the statistical analyses.

## Results

### Literature search

**Figure 1.**
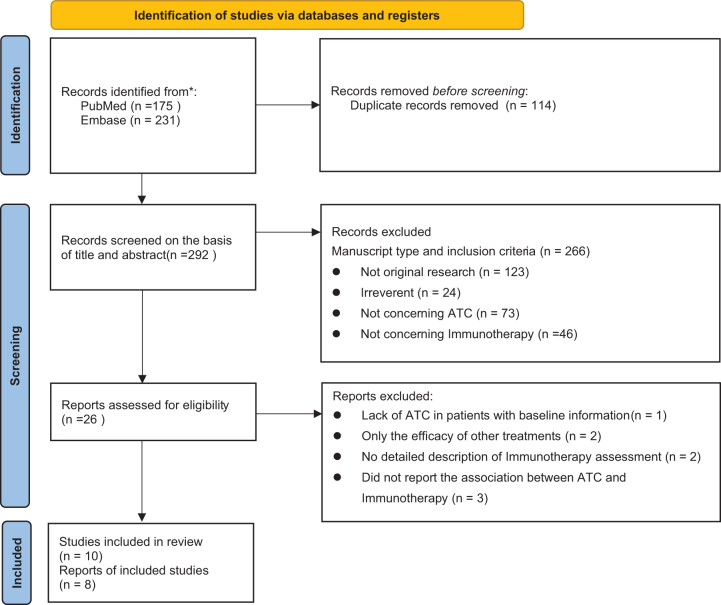
PRISMA diagram showing screening and selection of studies for systematic review and meta-analysis.

After initial searching, we identified 406 possibly relevant studies from the electronic databases. After removal of 114 duplicates, 292 studies were screened based on the abstract and title. Two hundred and sixty-six studies were excluded for various reasons (not original research (n = 123); irreverent (n = 24); not concerning the ATC (n = 73); not concerning immunotherapy (n = 46). Then, 26 studies were further excluded in full-text screening, of which 3 studies were without detailed information about the association between the ATC and immunotherapy, 2 studies were without a detailed description of immunotherapy assessment, 2 studies were without a clear definition of immunotherapy adherence, and 1 study only reported on a single component of ATC. In total, 18 studies (including 10 cohort studies and 8 case reports) met our eligibility requirements and were eventually included in this systematic review (Fig. 1).

### Baseline characteristics

Among 18 included studies, there were five prospective cohort studies, five retrospective cohort studies and eight case reports (Tables 1 and 2). The majority of the studies were performed in USA (n = 9)^[28,39–46]^, followed by Europe (n = 4)^[47–50]^ and Asia (n = 5)^[51–55]^. In cohort studies, the included patient sample sizes ranged from 3 to 55. The proportion of male patients across cohorts ranged from 20% to 66.7%, and the median age ranged from 51 to 70 years. The majority of ATC patients treated with immunotherapy in each cohort had distant metastases with clinical stage IVC. In the reported cohorts, more than half of the patients had PD-L1 expression of ≥1%. The proportion of ECOG≥1 varied considerably across cohorts, from 0 to 100%. About half of ATC patients in each cohort had BRAF^V600E^ mutation. Whether immunotherapy was the first treatment varied widely across cohorts, ranging from 40% to 100%. Patients in the cohort studies were followed up for 6-42 months.

**Table 1:** Characteristics of included cohort studies

Author (year)	Country	Study design	No of patient	Male n(%)	Median Age (yrs)	Stage IV			Distant metastasis	PD-L1 ≥ 1% n(%)	ECOG ≥1 n(%)	Genetic mutations	Naïve treatment	Treatment									
						A	B	C						Regimen	Follow-up(mos)	CR n(%)	PR n(%)	SD n(%)	PD n(%)	PFS (mos)	OS (mos)	1-year survival rate (%)	Adverse events (%)
Cabanillas (2024)	USA	Nonrandomized Clinical Trial	18	8 (44.4)	66	1	3	14	13 (72.2)	-	8 (44.4)	BRAF^V600E^: 100%	15 (83.3)	Atezolizumab + vemurafenib/cobimetinib	42.1	1 (5.6)	12 (66.7)	4 (22.2)	0	13.9	43.2	77.8	Colitis(3): 1, papilledema(3):1, retinopathy(1):1, pancreatitis(2):2.
			21	12 (57.1)	66	0	7	14	19 (90.1)	-	12 (57.1)	RAS/NF 100%	5 (23.8)	Atezolizumab + cobimetinib	8.7	0	3 (14.0)	3 (14.0)	9 (43.0)	4.8	8.7	42.9	Colitis(1): 1, pneumonitis(2):1 left ventricular dysfunction (3): 2.
			3	2 (66.7)	51	0	1	2	3 (100)	-	0	BRAF^V600E^/RAS/NF: 0	1 (33.3)	Atezolizumab + bevacizumab	6.2	0	1 (33.0)	0	2 (67.0)	1.4	6.2	0	Esophageal perforation(3):1
Tan (2024)	Singapore	Prospective	5	1 (20.0)	64	2	1	2	2 (40.0)	4 (80.0)	4 (80.0)	BRAF^V600E^: 80%	2 (40.0)	Radiotherapy + Pembrolizumab	32.6	2 (40.0)	2 (40.0)	0	1 (20.0)	7.6	-	40	Xerostomia(1):1,dry skin(1):1, anorexia(1):2,cough(1):1,breathless(1):1,giddiness(1):1fatigue(2):2, dysphagia(2):1,Laryngeal edema(3):1
Sehgal (2024)	USA	A Phase 2 Nonrandomized Clinical Trial	10	4 (40.0)	60.5	-	-	-	-	5 (62.5)	9 (90.0)	BRAF^V600E^: 30% NARS:40%	7 (70.0)	Nivolumab + Ipilimumab	22.2	0	3 (30.0)	2 (20.0)	4 (40.0)	4.3	-	55.6	-
Soll (2024)	Germany	Retrospective	5	3 (60.0)	65	0	1	4	4 (80.0)	5 (100)	2 (40.0)	BRAF^V600E^: 40% KRAS:20% TP53:20%	4 (80.0)	Lenvatinib + Pembrolizumab	25	0	1 (20.0)	3 (60.0)	0	4.7	6.3	20	Hypertension (2):1, fatigue (2):2, anorexia(2):2,anorexia(3):1,hand-foot-syndrome (1):1, diarrhea (1):1, proteinuria (1):4, abdominal pain (1):1,hemorrhages (3):1, tubulointer stitial nephritis (3):1, tracheoesophageal fistula (3):1, autoimmune hepatitis (3):1, pleural and pericardial effusion (1):1
Song (2024)	China	Retrospective	18	7 (38.9)	66	0	8	10	10 (55.6)	16 (88.9)	15 (83.3)	BRAF^V600E^: 50% BRAF wild type:50%	8 (44.4)	Lenvatinib/Anlotinib + PD-1 inhibitors	22	5 (27.8)	6 (33.3)	1 (5.6)	3 (16.7)	9.0	14	55.6	-
Wu (2023)	China	Retrospective	11	7 (63.6)	-	0	3	8	11 (100)	-	1 (9.1)	-	-	Targeted + Immunotherapy	24	-	-	-	-	5.5	20	73	-
			2	0	-	0	1	1	2 (100)	-	2 (100)	-	-	Immunotherapy	-	-	-	-	-	-	1	0	-
Hatashima (2022)	USA	Retrospective	13	7 (53.8)	70	4	2	7	9 (69.2)	8 (61.5)	10 (76.9)	BRAF^V600E^: 54%	3 (23.1)	Pembrolizumab or Nivolumab	13.5	1 (7.7)	2 (15.3)	3 (13.0)	5 (38.5)	1.9	3.9	38	Rash(1-2):2,endocrinopathies(1-2):3,(3-4):1,(5):1,nervous system(1-2):2,musculoskeletal (1-2):1,hypertension(1-2):5
Capdevila (2020)	Spain	Prospective	42	23 (54.8)	64	-	-	-	-	28 (70.0)	25 (59.5)	BRAF^V600E^: 28.6%	-	Spartalizumab	19.2	3 (7.0)	5 (12.0)	-	23 (57.0)	1.7	5.9	40	Diarrhea:5,pruritus:5,fatigue:3,Pyrexia:3,anemia:2,asthenia:2,myalgia:2,rash:2(all grades)
Chintakuntlawar (2019)	USA	A phase 2 one-sided study	3	1 (33.3)	56	0	3	0	0	-	3 (100)	-	-	Pembrolizumab + Chemoradiotherapy	6	0	0	0	0	-	2.76	0	-
Iyer (2018)	USA	Retrospective	12	8 (66.7)	60	0	3	9	9 (75.0)	10 (83.3)	10 (83.3)	BRAF^V600E^: 50%	5 (44.0)	Kinase inhibitor + pembrolizumab	8.14	0	5 (41.7)	4 (33.3)	3 (25.0)	2.96	6.93	42	-

BRAF, B-Raf proto-oncogene, serine/threonine kinase; CR, complete response; KRAS, Kirsten rat sarcoma viral oncogene homolog; mos, months; NARS, asparaginyl-tRNA synthetase; NF, neurofibromin; ORR, objective response rate; OS, overall survival; PD, progressive disease; PR, partial response; PFS, progression-free survival; RAS, rat sarcoma viral oncogene homolog; SD, stable disease; TP53, tumor protein P53; -, not applicable.

**Table 2:** Characteristics of included case reports

Author (year)	Country	Age (yrs)	Sex	Pre-treatment imaging examination	Stage IV	PD-L1	Genetic mutations	Previous treatment	Treatment						Follow-up		Quality of life after immunotherapy
									Systemic therapy	Combination therapy	Duration (mos)	Effects	Response	Adverse events	Time (mos)	Outcome	
Barbaro (2024)	Italy	65	Male	Left thyroid node (2 cm). Left cervical lymphadenopathy infiltrating with jugular vein and carotid artery wall.	B	(+)	BRAF^V600E^ (+) TERT^C250T^ (+) TP53^M246I^ (+)	Naive	Pembrolizumab + lenvatinib	Neoadjuvant surgery	1	Mass: mild reduction, colliquative degeneration. Carotid artery wall: free infiltration.	CR	-	12	No recurrence	-
Shih (2022)	China	58	Male	Thyroid mass with paratracheal and upper mediastinal invasion and bilateral pulmonary metastases.	C	-	-	Surgery	Pembrolizumab + doxorubicin + lenvatinib	Radiotherapy	2.7	Tumor decreased on the third and eighth week, leaving a tracheocutaneous fistula. New spinal metastases 8 weeks	PD	Nausea, vomiting, neutropenia, infection, pneumonia	2.7	Death	-
		71	Male	A diffusely enlarged thyroid gland. Left cervical lymphadenopathy. Multiple lung nodules.	C	-	-	None	Pembrolizumab + lenvatinib	None	1	Stationary neck tumor. Multiple enlarged pulmonary metastases, bronchovascular interstitial thickening.	PD	Dyspnea, dizziness, headache, pneumonia	1	Death	-
		59	Male	Thyroid mass invading prelaryngeal tissue, vocal cord, and thyroid cartilage. Bilateral neck lymphadenopathy.	B	-	-	Doxorubicin	Spartalizumab + doxorubicin	Debulking surgery	23	Extensive tumor necrosis, shrunken lymphadenopathy after 8 days. Tumor enlargement (4.4 cm) with fever and dysphagia after 23 months	PR	Infection	45.5	Alive	-
		60	Female	Cervical lymphadenopathy. Multiple pulmonary nodules.	C	(+)	BRAF^V600E^ (+)	Surgery, I131, sorafenib	Pembrolizumab + Lenvatinib/	Radiotherapy, Dabrafenib/trametinib	25 days	Stable pulmonary metastases. Neck tumor enlargement.	PD	Hepatitis, infection, pneumonia	18	Death	-
McCrary (2022)	USA	54	Male	A large 12 cm left thyroid mass Hypermetabolic cervical lymph nodes A 1.8 cm right lower lobe nodule concerning pulmonary metastasis	C	-	BRAF^V600E^ (-)	Surgery	Pembrolizumab + lenvatinib	Carboplatin AUC5 and paclitaxel	-	A significant reduction of the thyroid mass in both size and metabolic activity after 4 weeks	PR	G3 pure red cell aplasia additional clinical decompensation	-	Death	-
		59	Female	A large left thyroid mass invading the prevertebral fascia and esophagus	B	-	BRAF^V600E^ (-)	None	Pembrolizumab + lenvatinib	None	-	An early treatment response	PR	None	3	Alive	-
Ma (2022)	China	65	Male	ATC with brain and adrenal metastases	C	-	RET(+)	-	Cabozantinib + Nivolumab	Chemotherapy Palliative radiotherapy	30	Reduction of brain metastases	PR	-	-	Alive	-
Kroloff (2022)	USA	62	Female	A 6.5 cm ATC with extensive extra thyroidal extension Lymph node metastases	B	(+)	BRAF^V600E^ (+) TERT(+)	Surgery, radiotherapy	Nivolumab FS118	Radiotherapy, Dabrafenib and trametinib	36	A partial response, then progression of the necrotic parastomal mass in the left strap muscles after 7 months Rapid, subjective improvement in the neck mass along with improvement in swallowing	PR	High-grade fevers for 1 months, grade III drug-induced liver injury Nausea, fatigue, arthralgias	36	Alive	Remain on systematic therapy
Luongo (2021)	Italy	54	Female	Nodule 4.1 × 3.4 cm Multiple lymphadenopathies in the neck and mediastinum Lesion in the right inferior lobe(8.5 cm)	C	(-)	BRAF^V600E^ (-) NTRK(-) ALK(-)	Surgery	Pembrolizumab + Lenvatinib	Cisplatin + doxorubicin Paclitaxel Stereotactic radiotherapy	18	PR with a reduction by 50% of the sum of the diameters of the target lesions by RECIST A pleural nontarget lesion showed a progression	PR	Grade 3 diarrhea, vomiting, weight loss	18	Alive	Treatment still going
Cabanillas (2018)	USA	60	Male	An 8 cm left thyroid mass Tracheal deviation, significant lymphadenopathy	B	(+)	BRAF^V600E^ (+) TP53R175H(+) EGFRE322S(+)	Paclitaxel, carboplatin, trametinib	Dabrafenib + Trametinib + Pembrolizumab	Surgery, chemotherapy, radiotherapy	2	The nodules disappeared and the dermal metastases had completely resolved on exam and imaging	CR	None	11	Alive	Continuation of normal leisure activities such as hunting and golf
Kollipara (2017)	USA	62	Male	Nodule 3.4 × 3.0 cm Nodules in the right and left upper lobes of the lung	C	(+)	BRAF^V600E^ (+)	Surgery, I131, doxorubicin, cisplatin, paclitaxel,	Nivolumab	Vemurafenib	20	Regression of the right supraclavicular lymph node, decrease in the size of the previously documented pulmonary nodules Continued reduction of pulmonary lesions with complete radiographic resolution	PR	Progressive joint and bodyaches, exacerbation of psoriasis, nausea, vomiting, diarrhea, grade 2 colitis	20	Alive	-

ALK, anaplastic lymphoma kinase; BRAF, B-Raf proto-oncogene, serine/threonine kinase; CR, complete response; EGFR, epidermal growth factor receptor; mos, months; NTRK, neurotrophic tropomyosin receptor kinase; PD, progressive disease; PR, partial response; PTEN, phosphatase and tensin homolog; RET, rearranged during transfection; TP53, tumor protein; -, not applicable.

In eight case reports, a total of 11 patients were included (Table 2). The mean age of the patients was 60 years (age range, 50-71 years). Of these, seven were male and the remaining four were female. Clinical stage IVC accounted for 55%. PD-L1 was reported in six and positive in five. Mutations were reported in 9 cases, including six BRAF^V600E^ positive cases. Prior to immunotherapy, eight patients had received other treatments, mainly surgery. Imaging studies before immunotherapy showed lung metastases in 2 patients.

### Prognostic analysis of immunotherapy in cohort studies

The proportion of patients who responded well to immunotherapy (including CR, PR and SD) varied substantially across studies, ranging from 33% to 100%, reflecting considerable heterogeneity^[45,47,51,54]^. Besides, the 1-year survival rate of patients in the Cabanillas cohort (BRAF V600E mutation + triple therapy) was as high as 77.8%^[45]^, whereas other cohorts (e.g., Chintakuntlawar) report 0%^[28]^. The immunotherapy regimens used in different cohorts can be divided into three categories: (1) Use of immunotherapy drugs alone such as Spartalizumab^[48]^. (2) The combination of targeted drugs and immunotherapy drugs like Lenvatinib and Pembrolizumab^[46,47,56]^. (3) The form of application of radiotherapy and immunotherapy^[52,54]^. In general, combination treatment regimen showed better clinical efficacy, especially when ATC patients had BRAF^V600E^ gene mutation and corresponding targeted drugs such as Vemurafenib can significantly prolong the median survival time, up to 43.2 months^[45]^.

In the first clinical trial demonstrating the responsiveness of anaplastic thyroid cancer to PD-1 blockade^[51]^, patients with both BRAF non-mutant and mutant tumors experienced corresponding and durable immune responses. The median OS was 5.9 months (95% CI, 2.4 months to not reached), with 40% of patients surviving at 1 year. Notably, PD-L1–positive patients showed a significantly higher response rate (29%) compared to PD-L1–negative patients (0%), and their 1-year survival rate reached 52.1%. Similarly, the first non-RCT to report on a combination of anti-PD-1 and anti-CTLA-4 dual checkpoint inhibitors in advanced thyroid cancer found an ORR of 30%, with 50% of patients achieving clinical benefit. This was higher than the ORR of 19% in the previously mentioned single-agent trial^[39]^.

In addition to the application of immune checkpoint inhibitors, immunotherapy can also be combined with surgery or radiotherapy. In a case series of ATC patients treated with a combination of pembrolizumab and hypofractionated radiotherapy, two patients with prethyroidectomy and a non-BRAF-mutant phenotype had a complete response to treatment and remained disease-free at the last follow-up after discontinuation of treatment, and two patients had a partial response^[54]^. The 1-year survival rate and overall survival rate were both 40%. This indicates that immunotherapy combined with radiotherapy also has a certain effect on patients with non-BRAF mutation. Besides, the early resection of the thyroid to reduce the volume of the tumor may be more conducive to the combined treatment to target the residual tumor tissue or cells.

The results of other retrospective cohort studies suggest that immunotherapy may be an effective and well-tolerated treatment option for a subset of ATC patients, and that patients may derive clinical and survival benefits from immunotherapy combined with targeted therapy^[40,46,52,56]^. However, not all immunotherapies are effective. The initial efficacy data report for lenvatinib and pembrolizumab in patients with refractory ATC indicated that while most patients responded positively to the initial treatment, nearly all experienced disease progression within six months^[47]^. The OS for patients who did not respond to treatment was 6.3 months. Additionally, due to the typically small number of patients enrolled in studies of ATC immunotherapy, there may be an overemphasis on outliers, which complicates reliable comparisons.

### Single-proportion meta-analysis and publication bias

**Figure 2.**
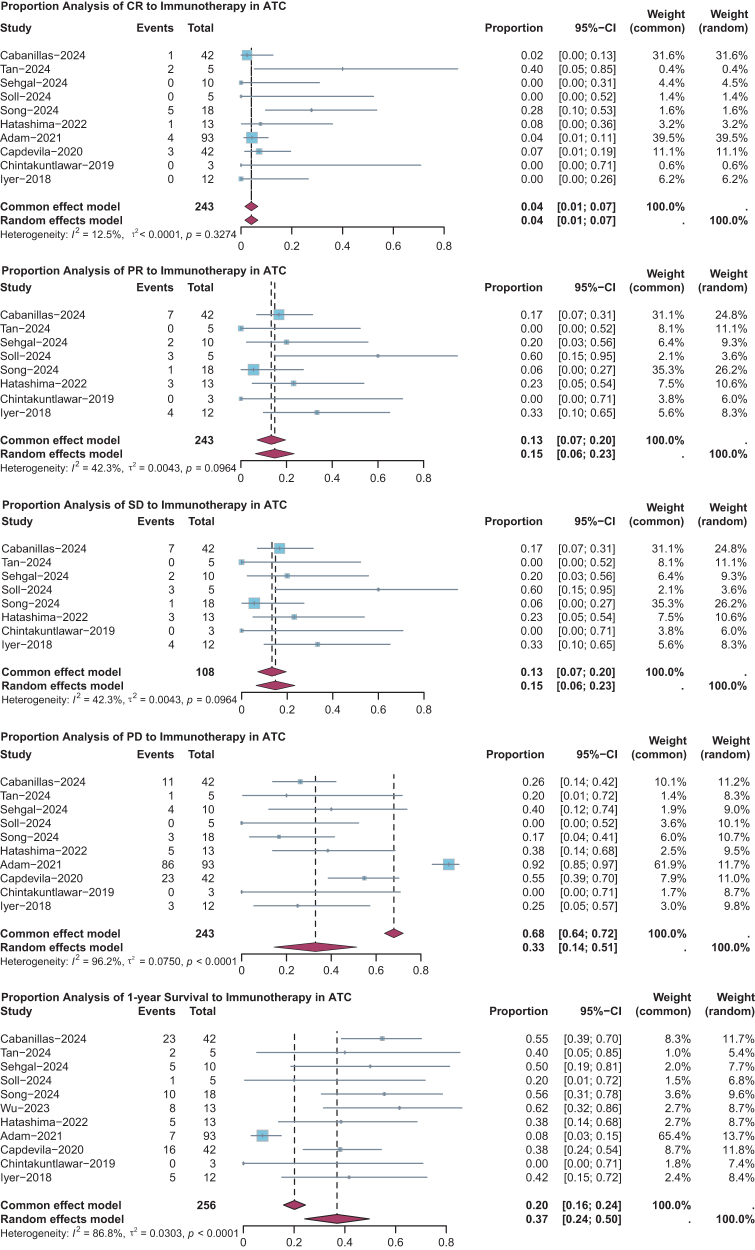
Forest plots showing the single-group rate meta-analysis within subgroups across various cohort studies. CR: complete response; PD: progressive disease; PR: partial response; PFS: progression-free survival; SD: stable disease.

**Figure 3.**
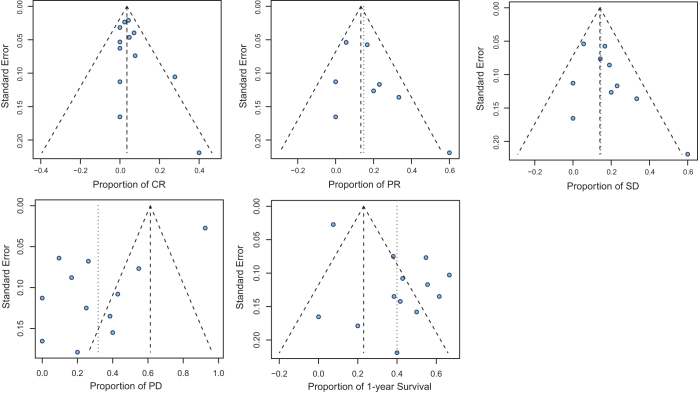
Funnel plots illustrating publication bias across different cohort studies within each subgroup. CR: complete response; PD: progressive disease; PR: partial response; PFS: progression-free survival; SD: stable disease.

To provide a more comprehensive assessment of the efficacy of immunotherapy in ATC, we conducted a single-proportion meta-analysis of CR, PR, SD, PD, and 1-year survival. Overall, the estimated proportions were as follows: CR 0.04 [0.01, 0.07], PR 0.15 [0.06, 0.23], SD 0.15 [0.06, 0.23], PD 0.33 [0.14, 0.51], and 1-year survival 0.37 [0.24, 0.50] (Fig. 2). Funnel plot analysis revealed significant publication bias in the PD and 1-year survival subgroups, while no significant bias was observed in the CR, PR, or SD groups (Fig. 3). Specifically, the PD funnel plot showed a left-skewed distribution, whereas the 1-year survival plot was skewed to the right, suggesting a publication tendency toward reporting higher survival rates and lower progression rates. This indicates that small-sample or lower-quality studies may have overestimated the efficacy of immunotherapy in reducing disease progression. Taken together, these findings suggest that immunotherapy provides clinical benefit for a subset of ATC patients (approximately one-third to one-half). However, its ability to effectively control disease progression and significantly improve 1-year survival remains limited.

**Figure 4.**
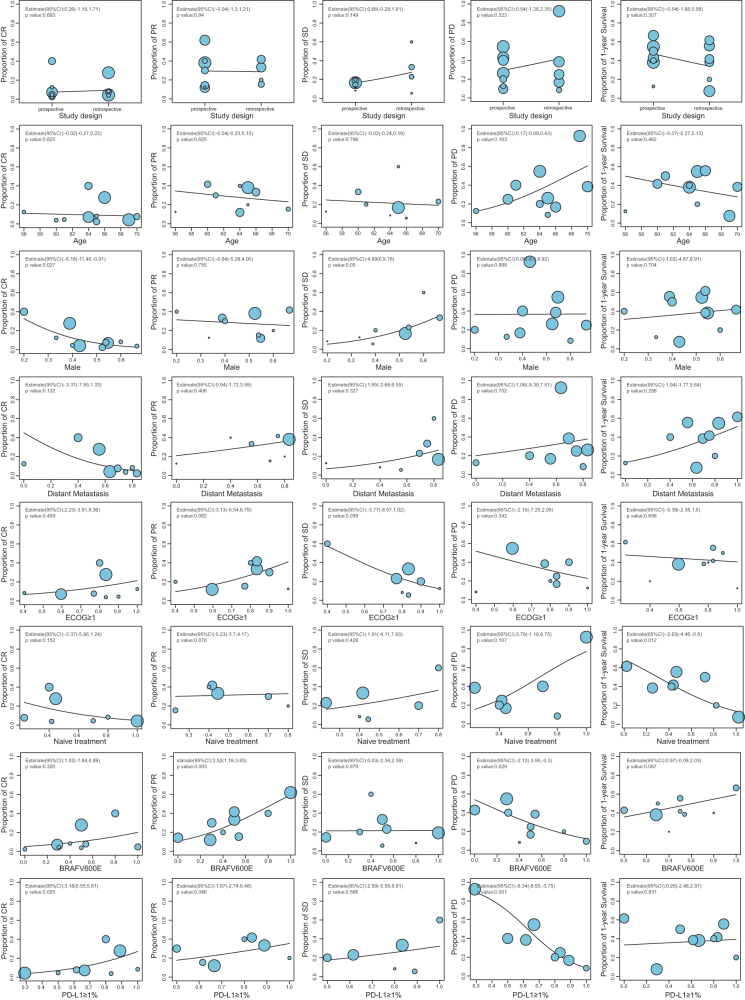
Bubble charts demonstrating the effect strength of different variables across subgroups. CR: complete response; PD: progressive disease; PR: partial response; PFS: progression-free survival; SD: stable disease.

To identify factors influencing the efficacy of immunotherapy in ATC, we conducted a meta-regression analysis based on single-arm proportions (Fig. 4). Variables such as study design, age, ECOG performance status, and the presence of distant metastasis showed no significant association with treatment outcomes. In contrast, cohorts with a higher proportion of PD-L1 ≥1% were associated with a higher probability of CR (3.18 [0.55, 5.81], *P* = 0.025) and a lower probability of PD (−6.34 [–8.93, −3.75], *P* = 0.001). Additionally, a higher proportion of BRAFV600E-mutant patients was significantly correlated with increased partial response (PR) rates (2.52 [1.19, 3.85], *P* = 0.003) and decreased PD rates (–2.12 [–3.95, −0.30], *P* = 0.029). Interestingly, a higher proportion of treatment-naïve patients within cohorts was paradoxically associated with lower 1-year survival rates.

### Prognostic analysis of immunotherapy in case reports

Among the eight case reports, two patients were completely cured after immunotherapy, and four patients died of disease progression after immunotherapy, among which 75% died of interstitial pneumonia caused by lung metastasis of tumors. In the first case of neoadjuvant therapy involving Lenvatinib and Pembrolizumab for patients with ATC and BRAF^V600E^ gene mutations, complete mass removal and a cure lasting at least one year were achieved. After one month of immunotherapy, the patient’s mass appeared visually softer and more mobile. A CT scan revealed almost complete colloid degeneration of the mass with no infiltration of the carotid wall. Following surgical resection of the mass, the patient was treated with I-131, and there was no recurrence during the subsequent one-year follow-up period^[49]^. In another case report on neoadjuvant therapy, a patient with a BRAF mutation in anaplastic thyroid cancer (ATC) was treated with pembrolizumab for immunotherapy at the time of disease progression. The patient experienced a partial response to the treatment, which may allow for complete surgical resection followed by postoperative chemoradiotherapy^[43]^. For the three patients who experienced adverse reactions, including interstitial pneumonia, infection, and respiratory failure due to lung metastasis, the thyroid tumor stabilized or shrank following immunotherapy. However, the lung metastasis exhibited signs of expansion and progression^[53]^. Therefore, immunotherapy should be carefully evaluated for ATC patients, particularly those with lung metastasis. It should be combined with other therapeutic modalities (such as targeted therapy, radiotherapy, etc.) to enhance efficacy. Additionally, it is essential to consider that since the majority of the case reports currently included are of moderate quality, with only one being of high quality, these reports likely overestimate efficacy.

### Side effects of immunotherapy in ATC

During immunotherapy, patients may experience various adverse reactions, including vomiting, diarrhea, fever, hypertension, and papilledema, among others. By categorizing these adverse events into grades 1 to 4, it becomes evident that the majority of patients in the included cohort studies experienced adverse events graded between 1 and 2. Only a minority of patients were rated as having grade 3 or 4 adverse events, such as urinary system manifestations including proteinuria and glomerulointerstitial nephritis, circulatory system issues like left ventricular dysfunction, hemorrhage, and edema. Digestive system manifestations include autoimmune hepatitis, esophageal perforation, colitis, and others. This data is derived from a retrospective cohort study published by Soll *et al*^[47]^, which indicates a high number of grade 3 adverse events (four), reflecting the modest clinical benefit of immunotherapy, with 60% of patients experiencing neither a response nor progression of their treatment. In another retrospective study published by Hatashima *et al*^[40]^, 38.5% of patients experienced disease progression, and even one patient suffered from grade 5 endocrinopathies, suggesting that changes in endocrine hormone indicators should be monitored during the use of immunotherapy to minimize the occurrence of adverse events.

Among the included case reports, two patients did not experience any adverse events following immunotherapy, which was consistent with the clinical benefit of partial response or complete cure. However, in the case report published by Shih *et al*^[55]^, three patients were infected with upper interstitial pneumonia and ultimately died of respiratory failure after undergoing immunotherapy. Since imaging examinations of these patients before immunotherapy indicated signs of lung metastasis, it suggests that immunotherapy may affect the immune balance of patients and increase the risk of infection. Overall, while mild adverse reactions during immunotherapy for ATC are inevitable, special attention should be given to life-threatening cardiopulmonary dysfunction caused by immune system dysregulation.

### Quality analysis of included studies

We used the NOS scale to score the included prospective cohort studies accordingly, and there were three high-quality studies (score 9), five medium-high-quality studies (score 7-8), and two medium-quality studies (score 4-6^[28,52,57]^) (Supplemental Digital Content Table 2, available at: http://links.lww.com/JS9/E956)^[33]^. In the quality assessment of case reports, we used the JBI assessment tool^[35]^, one high quality study (meeting 7 items), seven moderate quality studies (meeting 5-6 items^[42,44,45,49,50,53,55]^) (Supplemental Digital Content Table 3, available at: http://links.lww.com/JS9/F60).

## Discussion

ATC is the most malignant form of endocrine tumor, characterized by rapid growth and strong invasiveness. The majority of patients present with local invasion and distant metastasis at the time of diagnosis, resulting in an extremely poor prognosis. Currently, surgical intervention for ATC is challenging, and radiotherapy and chemotherapy remain the primary treatment modalities, albeit with limited effectiveness^[57]^. Consequently, investigating alternative treatment strategies, such as immunotherapy, is crucial to enhance therapeutic outcomes. This review indicates that while immunotherapy demonstrates some efficacy and can extend the survival rate for certain patients, particularly when used in conjunction with targeted therapy agents like dabrafenib for those with the BRAF^V600E^ mutation, the overall treatment efficacy has not yet achieved ideal results due to the significant heterogeneity across various studies. In clinical practice, immunotherapy should be selectively administered to patients who are more likely to respond based on their individual characteristics.

Meta-regression analysis identified PD-L1 expression, BRAF^V600E^ mutation, and treatment-naïve status as key factors associated with the efficacy of immunotherapy in ATC. Higher PD-L1 expression was significantly correlated with better treatment response, while patients with BRAF^V600E^ mutations showed improved outcomes when treated with a combination of BRAF-targeted therapy and immune checkpoint inhibitors. In contrast, a higher proportion of treatment-naïve patients was associated with poorer 1-year survival, possibly due to higher tumor burden at diagnosis or publication bias related to survival data reporting. Based on these findings, we propose the following treatment recommendations: (1) for patients with locally advanced, resectable disease, elective surgery should be considered following neoadjuvant immunotherapy; (2) for patients harboring BRAF^V600E^ mutations, combined therapy with BRAF inhibitors (e.g., dabrafenib) and immune checkpoint inhibitors (e.g., pembrolizumab) is recommended; (3) for other patients, the use of immunotherapy should be guided by PD-L1 expression levels.

Based on our inductive analysis, we suggest that immunotherapy should be used selectively in patients according to different grades of ATC and the expression of related genes (e.g., BRAF^V600E^) and antibodies. For patients with locally advanced disease who are eligible for surgery, elective surgical resection is recommended after neoadjuvant immunotherapy. For patients with BRAF^V600E^ mutation, the combination of BRAF mutation targeted drugs and immunotherapy drugs, such as Dabrafenib and Pembrolizumab, is recommended. For other patients, immunotherapy drugs should be used according to the expression of PD-L1. If the patient’s PD-L1 expression is positive, the corresponding immunosuppressants such as Pembrolizumab and Nivolumab can be used.

For the limited efficacy of immunotherapy in patients in some studies, we believe that there are three possible reasons. On the one hand, once ATC patients are diagnosed, their clinical grade is basically IVB or IVC stage, and ATC is very heterogeneous, which means that tumors from different patients may have different biological characteristics and genetic backgrounds, which may lead to different responses to immunotherapy^[58,59]^. At this time, the use of immunosuppressive drugs will make the situation of patients who have a history of underlying diseases or lung metastases even worse, leading to a series of more serious adverse events and even life-threatening patients. On the other hand, in terms of the choice of treatment plan, we found that the overall effect of the combination of targeted therapy and immunotherapy was better than that of radiotherapy and immunotherapy. For example, in a retrospective cohort study published by Chintakuntlawar *et al*, the combination of immunotherapy and radiotherapy and chemotherapy was used on patients, and the one-year survival rate of the patients was 0^[28]^. In other studies using targeted therapy and immunotherapy, the 1-year survival rate was as high as 77.8%.

Differences in the tumor immune microenvironment significantly influence the efficacy of immunotherapy in ATC. Single-cell RNA sequencing studies have shown that, compared to DTC, ATC exhibits a more immunosuppressive microenvironment characterized by T cell exhaustion, enrichment of cancer-associated fibroblasts, and M2-polarized macrophages^[60]^. The high proportion of exhausted T cells in ATC provides a rationale for anti-PD-1 therapies. However, the abundance of cancer-associated fibroblasts and M2 macrophages in ATC may contribute to immune evasion, angiogenesis, and metastasis, ultimately undermining the effectiveness of immunotherapy^[61,62]^. Emerging evidence highlights the importance of modulating the M1/M2 macrophage balance and targeting M2-like macrophages to enhance responses to PD-1/PD-L1 blockade and overcome resistance^[63]^. Therefore, immunotherapeutic strategies for ATC should not only focus on reinvigorating T cells but also consider targeting macrophages and fibroblasts to improve treatment outcomes.

In addition, immune-related adverse events may involve multiple organ systems, including thyroid dysfunction (e.g., thyroiditis, hypothyroidism), skin toxicity (e.g., rash, pruritus), gastrointestinal reactions (e.g., diarrhea), pulmonary inflammation (e.g., pneumonia), and endocrine dysfunction^[64–66]^. Among them, pneumonia is a potentially fatal disease, with pulmonary symptoms usually manifested as cough, shortness of breath, chest pain, varying degrees of dehydration and fever, eventually causing infection or respiratory failure leading to death^[67–69]^. For ATC patients, the cause of checkpoint inhibitor-associated pneumonia may be related to its mediated immunosuppression mechanism^[70]^. It is well known that immunosuppressants enhance the activity of T cells by blocking immunosuppressive signals, such as PD-1/PD-L1 or CTLA-4, leading to increased antitumor efficacy. However, the excessive activation of non-specific immunity may lead to the destruction of the balance between the subsets of T cells and the abnormal expression of other inflammatory factors, which eventually leads to the attack of normal lung tissue and causes an inflammatory response^[71,72]^.

Therefore, precise management strategies are needed for the various types of adverse events that may occur in ATC patients. Additionally, immunosuppressive cells and metabolic by-products in the tumor microenvironment may also limit the effectiveness of immunotherapy^[73]^. Based on this, future immunotherapy-related research on ATC should focus on understanding the impact of the immune microenvironment on treatment outcomes, optimizing immunotherapy strategies, and exploring the potential of combination therapies to provide more effective treatment options. Considering the differences in tumor microenvironment and immune status of different patients, different personalized treatment regimens also need to be developed.

Several limitations should also be acknowledged in this review. Firstly, the included criteria were limited to English language studies, potentially leading to exclusion of non-English relevant data and publication bias. Secondly, despite conducting a single proportion meta-analysis, there remains high heterogeneity among different studies within each subgroup, which may be attributed to variations in study design types, differences in gene expression, disparities in sample sizes, and other influencing factors.

## Conclusion

In summary, numerous studies on ATC related to immunotherapy have, to some extent, confirmed its safety and efficacy in patients, particularly the synergistic effect when used in conjunction with other therapies such as chemotherapy or targeted therapy. Consequently, neoadjuvant therapy is recommended for patients with locally advanced disease who are candidates for surgery. For patients with the BRAF^V600E^ mutation, the combination of targeted drugs and immunotherapy is advised. Besides, the management of side effects should be strengthened, especially to prevent the occurrence of interstitial pneumonia. However, due to the limited availability of relevant research literature and the small number of ATC patients included in this study, a comprehensive evaluation of the efficacy and applicability of immunotherapy across different ATC subtypes remains challenging. Therefore, further large-scale, multicenter prospective studies are needed to identify ATC patient populations that are sensitive to immunotherapy.

## Supplementary Material

## Data Availability

All relevant data supporting the findings of this study are available within the supplementary materials and/or upon request. The datasets generated and/or analyzed during the current study are available from the corresponding author on reasonable request.

## Research registration unique identifying number (UIN)

The study has been registered with the International Prospective Register of Systematic Reviews (PROSPERO: https://www.crd.york.ac.uk/prospero/) prior to the initiation of data extraction (CRD42024586589).
