# Supplementary material for: Efficacy and safety of immunotherapy in anaplastic thyroid carcinoma: a systematic review and meta-analysis
Source: Int J Surg. 2025 Aug 27;112(1):1708–21. doi: 10.1097/JS9.0000000000003301 (PMC12825863; doi:10.1097/JS9.0000000000003301)
Supplement: Supplementary file 1 [file js9-112-1708-001.docx]

**Table S1A. The search strategy used in the meta-analysis conducted in PubMed. (From inception to Dec 31, 2024)**

| **PubMed** |  | **Number** |
| --- | --- | --- |
| #1 | “Anaplastic thyroid carcinoma”[MeSH Terms] | 1129 |
| #2 | “thyroid carcinoma, anaplastic” OR (“thyroid”AND“carcinoma” AND "anaplastic” ) OR “anaplastic thyroid carcinoma” OR (“anaplastic” AND “thyroid” AND “carcinoma” ) | 4084 |
| #3 | “Poorly differentiated thyroid carcinoma”[MeSH Terms] | 1427 |
| #4 | “Poorly” AND (“cell differentiation” OR (“cell"AND “differentiation” ) OR “cell differentiation” OR “differentiated" OR “differentiation” OR “differential” OR “differentials” OR “differentiate” OR “differentiates” OR “differentiating” OR “differentiational” OR “differentiations” OR “differentiative” ) AND (“thyroid neoplasms” OR (“thyroid” AND “neoplasms” ) OR “thyroid neoplasms” OR (“thyroid” AND “carcinoma” ) OR “thyroid carcinoma” ) | 1929 |
| #5 | #1 OR #3 | 2469 |
| #6 | “Immunotherapy”[MeSH Terms] | 355536 |
| #7 | “immunotherapy”[MeSH Terms] OR “immunotherapy” OR “immunotherapies” OR “immunotherapys” OR“immune checkpoint inhibitor” | 466161 |
| #8 | #1 AND #6 | 28 |
| #9 | #2 AND #7 | 135 |
| #10 | #3 AND #6 | 9 |
| #11 | #4 AND #7 | 33 |
| #12 | #5 AND #6 | 32 |
| #13 | #12 filters: humans, English | 30 |

**Table S1B. The search strategy used in the meta-analysis conducted in Embase. (From inception to Dec 31, 2024)**

| **Embase** |  | **Number** |
| --- | --- | --- |
| #1 | ‘Anaplastic thyroid carcinoma’/exp | 3436 |
| #2 | 'anaplastic thyroid carcinoma’ OR 'thyroid carcinoma，anaplastic' OR ‘atc’ OR ‘undifferentiated thyroid carcinoma’ | 10701 |
| #3 | #1 OR #2 | 12199 |
| #4 | ‘Poorly differentiated thyroid carcinoma’/exp | 1129 |
| #5 | ‘poorly differentiated thyroid carcinoma’ OR ‘differentiated thyroid carcinoma，poorly’ OR ‘pdtc’ OR ‘celll differentiation’ OR ‘poorly differential thyroid carcinoma’ OR ‘differentiation thyroid carcinoma’ OR ‘differentials thyroid carcinoma’ OR ‘differentiative thyroid carcinoma’ | 3595 |
| #6 | #4 OR #5 | 4228 |
| #7 | ‘Immunotherapy’/exp | 356251 |
| #8 | ‘immune therapy’ OR‘ immunotherapy’ OR ‘immune checkpoint inhibitor’ OR ‘immune checkpoint inhibitor therapy’OR ‘cancer immunotherapy’ OR ‘immunotherapys’ | 246304 |
| #9 | #7 OR #8 | 428568 |
| #10 | #3 AND #9 | 460 |
| #11 | #6 AND #9 | 71 |
| #12 | #3 OR #6 | 15851 |
| #13 | #12 AND #9 | 491 |
| #14 | #13 AND [english]/lim | 239 |
| #15 | #14 AND 'human'/de | 240 |
