# Supplementary material for: Efficacy and safety of immunotherapy in anaplastic thyroid carcinoma: a systematic review and meta-analysis
Source: Int J Surg. 2025 Aug 27;112(1):1708–21. doi: 10.1097/JS9.0000000000003301 (PMC12825863; doi:10.1097/JS9.0000000000003301)
Supplement: Supplementary file 2 [file js9-112-1708-002.docx]

**Supplementary Table 2. The quality of included cohort studies assessed by the Newcastle-Ottawa Quality Assessment Scale.**

| **Category** | **Item** | **Option** | **Chintakuntlawar**  **(2019)** | **Capdevila**  **(2020)** | **Tan**  **(2024)** | **Cabanillas**  **(2024)** | **Sehgal**  **(2024)** | **Iyer**  **(2018)** | **Hatashima**  **(2022)** | **Wu**  **(2023)** | **Soll**  **(2024)** | **Song**  **(2024)** |
| --- | --- | --- | --- | --- | --- | --- | --- | --- | --- | --- | --- | --- |
| Selection | Representativeness of the exposed cohort | Truly representative of the general population* | N | N | N | N | N | N | N | N | N | N |
|  |  | Somewhat representative of the general population* | N | Y* | Y* | Y* | Y* | Y* | Y* | Y* | Y* | Y* |
|  |  | Selected group of users eg nurses, volunteers | N | N | N | N | N | N | N | N | N | N |
|  |  | No description of the derivation of the cohort | N | N | N | N | N | N | N | N | N | N |
|  | Selection of the non exposed cohort | Drawn from the same community as the exposed cohort* | Y* | Y* | Y* | Y* | Y* | Y* | Y* | Y* | Y* | Y* |
|  |  | Drawn from a different source | N | N | N | N | N | N | N | N | N | N |
|  |  | No description of the derivation of the non-exposed cohort | N | N | N | N | N | N | N | N | N | N |
|  | Ascertainment of exposure | Secure record (i.e. medical records incl. lab results, or blood measures)* | Y* | Y* | Y* | Y* | Y* | Y* | Y* | N | Y* | Y* |
|  |  | Structured interview* | N | N | N | N | N | N | N | N | N | N |
|  |  | Written self-report | N | N | N | N | N | N | N | N | N | N |
|  |  | No description | N | N | N | N | N | N | N | N | N | N |
|  | Demonstration that outcome of interest was not present at start of study/before as certainment of exposure | Yes,if individuals with outcome of interest diagnosed/reported within the first year after study entry/ascertainment of exposure are excluded or considered as non-exposed* | N | Y* | Y* | Y* | Y* | N | N | N | N | N |
|  |  | No, otherwise | N | N | N | N | N | Y | Y | Y | Y | Y |
| Comparability | Comparability of cohorts on the basis of the design or analysis | Study controls for the most important factor* | Y* | Y* | Y* | Y* | Y* | N | Y* | Y* | Y* | N |
|  |  | Study controls for any additional factor (This criteria could be modified to indicate specific control for a second important factor.) * | Y* | Y* | Y* | Y* | Y* | Y* | Y* | N | Y* | Y* |
| Outcome | Assessment of outcome | Independent blind assessment (e.g.national/regional cancer registry) assessment (including radiology/histology/anapath.confirmation) | Y | Y | Y | Y | Y | Y | Y | Y | Y | Y |
|  |  | Record linkage* | Y* | Y* | Y* | Y* | Y* | Y* | Y* | Y* | Y* | Y* |
|  |  | Self-report (i.e. no reference to original medical records or x-rays to confirm the outcome) | N | N | N | N | N | N | N | N | N | N |
|  |  | No description | N | N | N | N | N | N | N | N | N | N |
|  | Was Follow-Up Long Enough for Outcomes to Occur | Yes, it is an adequate follow up period for outcome of interest* | N | Y* | N | Y* | Y* | Y* | Y* | Y* | Y* | Y* |
|  |  | No | Y | N | Y | N | N | N | N | N | N | N |
|  | Adequacy of follow up of cohorts | Complete follow up - all subjects accounted for * | N | N | N | N | N | Y* | N | Y* | N | N |
|  |  | Subjects lost to follow up unlikely to introduce bias - small number lost - >80 % (select an adequate %) follow up, or description provided of those lost) * | N | Y* | Y* | Y* | Y* | N | Y* | N | N | Y* |
|  |  | Follow up rate < 80% (select an adequate %) and no description of those lost | Y | N | N | N | N | N | N | N | Y | N |
|  |  | No statement | N | N | N | N | N | N | N | N | N | N |
| Quality Score | | | 5 | 9 | 8 | 9 | 9 | 7 | 8 | 6 | 7 | 7 |
