# Supplementary material for: Efficacy and safety of immunotherapy in anaplastic thyroid carcinoma: a systematic review and meta-analysis
Source: Int J Surg. 2025 Aug 27;112(1):1708–21. doi: 10.1097/JS9.0000000000003301 (PMC12825863; doi:10.1097/JS9.0000000000003301)
Supplement: Supplementary file 3 [file js9-112-1708-003.docx]

**Supplementary Table 3. The quality of included case reports assessed by the Joanna Briggs Institute.**

| Item | Kollipara  (2017) | Cabanillas  (2018) | Luongo  (2021) | Kroloff  (2022) | Ma  (2022) | McCrary  (2022) | Shih  (2022) | Barbaro  (2024) |
| --- | --- | --- | --- | --- | --- | --- | --- | --- |
| Whether the patient's demographic characteristics are clearly described | NC | NC | NC | NC | NC | NC | NC | NC |
| Whether the patient's history is clearly described in chronological order | Y | Y | Y | Y | NC | NC | Y | NC |
| Whether the patient's current clinical presentation is clearly described | Y | Y | Y | Y | Y | Y | Y | Y |
| Whether diagnostic or assessment methods and results are clearly described | Y | Y | Y | Y | Y | Y | Y | Y |
| Whether the intervention or treatment is clearly described | Y | Y | Y | Y | Y | Y | Y | Y |
| Whether the post-treatment clinical presentation is clearly described | Y | Y | Y | Y | Y | Y | Y | Y |
| Whether adverse effects or unexpected events were identified and described | Y | NA | NC | Y | NA | NC | Y | NA |
| Whether it provides a reference to the experience | NC | NC | Y | Y | Y | Y | NC | Y |

Y:Yes N:No NC:Not Clear NA:Not Applicable
